# Association between social participation and mental health consultation in individuals with suicidal ideation: a cross-sectional study

**DOI:** 10.1186/s12888-020-02724-8

**Published:** 2020-06-16

**Authors:** Hin Moi Youn, Soo Hyun Kang, Sung-In Jang, Eun-Cheol Park

**Affiliations:** 1 Department of Public Health, Graduate School, Yonsei University, Seoul, Republic of Korea; 2 Institute of Health Services Research, Yonsei University, Seoul, Republic of Korea; 3 Department of Preventive Medicine, Yonsei University College of Medicine, Seoul, Republic of Korea

**Keywords:** Social participation, Mental health consultation, Suicide prevention, Suicide, Suicidal behavior, Suicidal ideation

## Abstract

**Background:**

Suicidal ideation is a significant public health concern worldwide. Although suicides might be preventable through the provision of adequate treatment, mental health consultation is still mostly underutilized. This study thus aimed to examine the association between social participation and utilization of mental health consultations in individuals with suicidal ideation.

**Methods:**

Data were collected from the nationwide Community Health Survey (conducted by the Korea Centers for Disease Control and Prevention, 2017). A total of 17,067 individuals (men: 32.9%, women: 67.1%) who reported experiencing suicidal ideation were included in the analysis. The mean age of the study population was 60.1 (±17.8) years old. This study examined social participation; the number of social activities participated in among leisure, volunteer, social, and religion related activities. Multivariate logistic regression was then used to assess the significance of these associations.

**Results:**

Among those experienced suicidal ideation, 1860 (10.9%) reported receiving mental health consultation services (men: 8.8%, women: 11.9%). Overall, an increased social participation was significantly associated with increased odds of using forms of mental health consultation (OR = 1.65, 95% CI: 1.31–2.09).

**Conclusions:**

In this study, significant evidence of the links between social participation and utilization of mental health consultation was discovered among at risk individuals with suicidal ideation. Suicide prevention policies and programs designed to enhance social participation could potentially encourage people at suicide risk to seek the help they need. Further research focusing on social approaches can produce useful information to plan and implement comprehensive and effective strategies.

## Background

Suicidal behavior is a global public health concern. Suicides account for 1.4% of all deaths worldwide, making it the 18th leading cause of death in 2016 [1]. Korea has had one of the highest suicide rates over the last several decades when compared to other members of the Organization for Economic Cooperation and Development (OECD) [2]. As of 2018, the suicide rate in Korea increased by 9.5%, meaning that it was the cause of 26.6 per 100,000 deaths [3].

Suicidal behaviors, including suicidal ideation, plans, and attempts, are strongly predictive of death by suicide. Although suicide prevention requires a comprehensive and multidisciplinary approach, one of the well-known and efficacious interventions in reducing suicide risks is ensuring that people at risk receive appropriate treatment for their mental health problems [4]. However, despite the increased need for professional services, the vast number of people at risk for suicide are not in treatment [5, 6]. Individuals who fail to receive adequate treatment may experience an escalation of symptoms, such as the progression of depression into suicidal behaviors [7]. Therefore, enhancing engagement in treatment through mental health services may be one of the key strategies in preventing suicidal behaviors and death. As a result, numerous studies have been conducted to better understand help-seeking and mental health service utilization among suicidal individuals and to identify contributing factors that influence individuals’ decisions to utilize mental health services [8–10]. These studies have ranged in their scope and focus, and some have been aimed at social factors [11].

Social participation, as well as active engagement in volunteering and religious activities, are found to be associated with better mental and physical health and well-being [12–14]. Furthermore, individuals’ pathway into treatment are often influenced by the support provided by their social contacts [15]. However, social participation or other forms of social factors do not always lead to mental health services utilization. Some studies have presented possible effects of social factors on mental health help-seeking behaviors [16–19]. Gourash outlined four hypothetical avenues concerning the interactions between a person’s social networks and their mental health service utilization: buffering the experience of stress, therefore obviating the need for help; providing instrumental and emotional support that substitutes for professional assistance; advocating for or referring to services; and transmitting attitudes, values, and norms about the nature of help-seeking behaviors [19].

This study sought to examine the association between social participation, or the social activities that individuals at risk of suicidal behaviors engage in regularly, and their utilization of mental health consultation.

## Methods

### Study population

This study used data from the Korean Community Health Survey (KCHS), a nationwide cross-sectional survey. The KCHS is designed to collect data to plan, implement, monitor, and evaluate community-level health promotion and disease prevention programs by the Korea Centers for Disease Control and Prevention (KCDC). The KCHS has been conducted annually since 2008 by trained interviewers through direct face-to-face interviews, and 253 national public health centers have participated. The survey subjects are aged 19 or older were selected by the probability proportional sampling method and the systematic sampling method. The data consisted of 358 questionnaires regarding sociodemographic characteristics, disease and relevant information, life style, and health behavior information. The KCHS is annually reviewed and approved by the institutional review board of the KCDC, and written informed consent is obtained from all participants. The data is freely accessible online at [http://chs.cdc.go.kr]. In 2017, among 228,381 individuals, 17,450 (7.6%) reported having experienced suicidal ideation only, and 740 (0.3%) reported having experienced both suicidal ideation and suicide attempt within 1 year. In the study, we included those who had suicidal ideation only. Suicidal ideation was measured by asking whether the subject had thoughts of suicide within the past year [20]. The questions were assessed using “yes” or “no” answers based on the subjects’ self-report. Data for any subjects with missing values for other study variables were excluded. In the end, a final sample population of 17,067 who had experienced suicidal ideation within the past year was utilized.

### Variables

#### Mental health consultation utilization

The dependent variable in this study was subjects’ use of mental health consultation services regarding their suicidal ideation. Subjects who reported having suicidal ideation within 1 year were asked the following question, “Did you receive any mental health consultation services from a medical institution, professional consulting institution, or local mental health center in regards to your suicidal ideation?” In the study, mental health consultation service use was determined by “yes” or “no” responses.

#### Social participation

The main independent variable of interest in this study is social participation. Social participation was determined as the number of social activities in which a subject participated within 1 year. Options included leisure, voluntary, religious, and social gatherings. Participants could respond with no activities (0), one activity (1), two activities (2), and more than three activities (3). The study also measured the frequency of social contact with family, neighbors, and friends (less than once per week, more than once per week).

#### Demographic characteristics and health status

Covariates regarding participants’ demographic characteristics included their sex, age (grouped as 19–29 years, 29–39 years, 40–49 years, 50–59 years, 60–69 years, 70–79 years, or ≥ 80 years), education level (≤middle school graduate, high school graduate, college graduate, or ≥ university graduate), occupation (unemployed/housewives/soldiers/students, low-skilled blue collar, high-skilled blue collar, low-skilled white collar, or high-skilled white collar), marital status (no spouse or with spouse), household income (≤1 million won/month, 1–2 million won/month, 2–3 million won/month, 3–4 million won/month, or ≥ 4 million won/month), number of household members (1, 2, or ≥ 3), region (rural, urban, or metropolitan), and basic livelihood security recipient status (yes or no). Health-related covariates included the presence of depressive symptoms (yes or no), perceived health status (unhealthy, average, or healthy), difficulties in daily routine activities (yes or no), currently smoking (yes or no), and high risk drinking (yes or no). The study also included main reasons for suicidal ideation (physical problems such as illnesses or disability, financial difficulties, loneliness, domestic troubles, difficulties at work, or others such as problems with romantic relationships and concerns about career building).

### Statistical analysis

All analyses included the use of weighted variables. Descriptive analysis was conducted first, followed by logistic regression analysis, to determine the odds ratios (ORs) and confidence intervals (95% CIs) using “proc survey logistic.” For the logistic regression analysis, the association between social participation and mental health consultation was assessed for those who experienced suicidal ideation. Statistical analyses were performed using SAS software version 9.4 (SAS Institute, Cary, NC, USA).

## Results

**Fig. 1:**
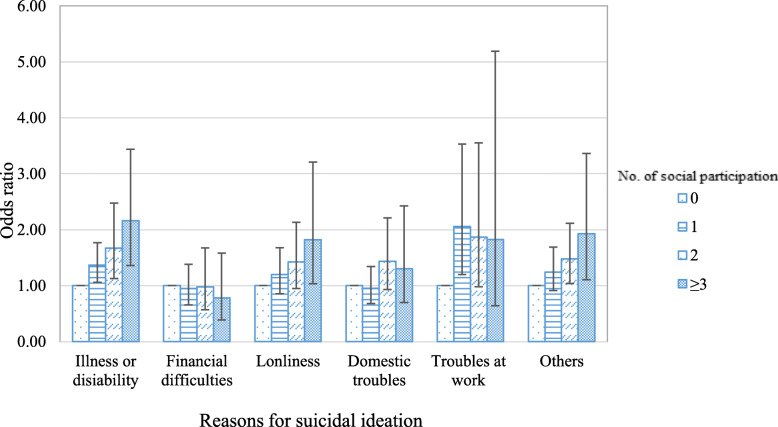
Subgroup analysis of mental health consultation according to number of social pariticipation stratified by reasons for suicidal ideation

The general characteristics of the study population are summarized in Table 1. Among the 17,067 individuals in the study population, 1860 (10.9%) received mental health consultation, whereas 15,207 (89.1%) did not. More than half of individuals (56.0%) participated regularly in one or more social activities, while 44% did not participate in any social activities. Of those who reported participation in at least one social activity, 11.6% received mental health consultation for their suicidal thoughts, whereas 10.0% of those who did not have any social participation received mental health consultation. Individuals had social contact more than once a week with family (54.6%), friends (43.1%), and neighbors (57.3%). In total, physical health problems such as illness or disabilities accounted for 30.7%, followed by financial difficulties (17.2%), other reasons (17.2%), loneliness (15.2%), and domestic troubles (14.2%). In the study, more women (67.1%) reported experiencing suicidal ideation than men (32.9%), and the proportion of mental health consultation use was slightly higher in women (11.9%) than men (8.8%) as well. Table 2 shows the results of logistic regression analyzing the association between social participation and mental health consultation. The results showed that increased numbers of social activities were significantly associated with increased odds of using mental health consultation. Those who participated in more than three social activities had the highest odds for using mental health consultation (OR = 1.67, 95% CI: 1.20–2.32) compared to individuals with no social participation. The frequency of social contact with family, friends, or neighbors did not show a significant effect. Individuals who thought about suicide due to financial difficulties were the least likely to use mental health consultation (OR = 0.65, 95% CI: 0.52–0.80), and individuals whose main reason for suicidal ideation was domestic troubles were the most likely to use mental health consultations (OR = 1.36, 95% CI: 1.11–1.68). Table 3 shows the analysis results regarding the association between social participation and mental health consultation according to types of social activities. Individuals who participated in leisure/sports (OR = 1.47, 95% CI: 1.25–1.73) and religion (OR = 1.17, 95% CI: 1.02–1.33) related activities showed significantly higher odds of using mental health services compared to those with no social participation. Figure 1 presents the results for the subgroup analysis of the association between social participation and mental health consultation, stratified by the main reasons for having suicidal ideation. The analysis reveals that respondents whose reasons were related to physical health problems such as illness or disabilities showed a graded positive association (more than three: OR = 2.16, 95% CI: 1.36–3.44).

**Table 1:** General characteristics of the study population

Variables	Mental health consultation						
	Total		Yes		No		***P***
	N	%	N	%	N	%	
	17,067	100.0	1860	10.9	15,207	89.1	
**No. of social participation**^**a**^							0.0553
0	7511	44.0	753	10.0	6758	90.0	
1	5905	34.6	647	11.0	5258	89.0	
2	2741	16.1	324	11.8	2417	88.2	
≥ 3	910	5.3	136	14.9	774	85.1	
**Frequency of social contact with family**							0.5935
Less than once/w	7744	45.4	869	11.2	6875	88.8	
More than once/w	9323	54.6	991	10.6	8332	89.4	
**Frequency of social contact with friends**							0.2882
Less than once/w	9710	56.9	1031	10.6	8679	89.4	
More than once/w	7357	43.1	829	11.3	6528	88.7	
**Frequency of social contact with neighbor**							0.0037
Less than once/w	7291	42.7	923	12.7	6368	87.3	
More than once/w	9776	57.3	937	9.6	8839	90.4	
**Reasons for suicidal ideation**							<.0001
Illness or disability	5236	30.7	500	9.5	4736	90.5	
Financial difficulties	2928	17.2	253	8.6	2675	91.4	
Loneliness	2594	15.2	312	12.0	2282	88.0	
Domestic troubles	2418	14.2	332	13.7	2085	86.2	
Troubles at work	961	5.6	117	12.2	844	87.8	
Others	2930	17.2	346	11.8	2584	88.2	
**Sex**							<.0001
Male	5611	32.9	496	8.8	5115	91.2	
Female	11,456	67.1	1364	11.9	10,092	88.1	
**Age**							<.0001
19–29	1138	6.7	219	19.2	919	80.8	
29–39	1503	8.8	226	15.0	1277	85.0	
40–49	2121	12.4	295	13.9	1826	86.1	
50–59	2934	17.2	351	12.0	2583	88.0	
60–69	3149	18.5	378	12.0	2771	88.0	
70 ~ 79	3805	22.3	301	7.9	3504	92.1	
≥ 80	2417	14.2	90	3.7	2327	96.3	
**Educational level**							<.0001
None/Middle school graduate	9389	55.0	799	8.5	8590	91.5	
High school graduate	4190	24.6	557	13.3	3633	86.7	
College graduate	1165	6.8	136	11.7	1029	88.3	
University graduate/higher	2323	13.6	368	15.8	1955	84.2	
**Occupation**							<.0001
Others^b^	9142	53.6	1061	11.6	8081	88.4	
Low-skilled blue collar	2627	15.4	244	9.3	2383	90.7	
High-skilled blue collar	1965	11.5	149	7.6	1816	92.4	
Low-skilled white collar	1727	10.1	197	11.4	1530	88.6	
High-skilled white collar	1606	9.4	209	13.0	1397	87.0	
**Marital status**							0.0002
W/o spouse	7388	43.3	848	11.5	6540	88.5	
With spouse	9679	56.7	1012	10.5	8667	89.5	
**Number of household members**							0.4388
1	4208	24.7	400	9.5	3808	90.5	
2	6520	38.2	706	10.8	5814	89.2	
≥ 3	6339	37.1	754	11.9	5585	88.1	
**Region**							<.0001
Rural	6018	35.3	499	8.3	5519	91.7	
Urban	6718	39.4	779	11.6	5939	88.4	
Metropolitan	4331	25.4	582	13.4	3749	86.6	
**Household Income**							0.6977
Less than 1 million won/m	6821	40.0	658	9.6	6163	90.4	
1–2 mil won/m	3071	18.0	340	11.1	2731	88.9	
2–3 mil won/m	2509	14.7	301	12.0	2208	88.0	
3–4 mil won/m	1776	10.4	198	11.1	1578	88.9	
Over 4 mil won/m	2890	16.9	363	12.6	2527	87.4	
**Basic livelihood security recipient**							<.0001
Yes	1618	9.5	271	16.7	1347	83.3	
No	15,449	90.5	1589	10.3	13,860	89.7	
**Depressive symptoms**							<.0001
Yes	6216	36.4	1191	19.2	5025	80.8	
No	10,851	63.6	669	6.2	10,182	93.8	
**Perceived health status**							<.0001
Unhealthy	8701	51.0	1012	11.6	7689	88.4	
Average	5823	34.1	612	10.5	5211	89.5	
Healthy	2543	14.9	236	9.3	2307	90.7	
**Difficulty in daily activity**							0.4020
Yes	6078	35.6	605	10.0	5473	90.0	
No	10,989	64.4	1255	11.4	9734	88.6	
**Currently Smoking**							0.7938
Yes	3026	17.7	344	11.4	2682	88.6	
No	14,041	82.3	1516	10.8	12,525	89.2	
**High risk drinking**							0.0871
Yes	1814	10.6	175	9.6	1639	90.4	
No	15,253	89.4	1685	11.0	13,568	89.0	

^a^ Number of social activities a respondent is currently participated in among religion, social, leisure and volunteer activities

^b^ Others include housewives, students, soldiers and unemployed

**Table 2:** Logistic regression analysis to investigate the association between social participation and mental health consultation

Variables	Mental health consultation				
	OR	95% CI			***P***
**No. of social participation**^**a**^					
0	1.00				
1	1.22	(1.07	–	1.40)	0.2305
2	1.41	(1.18	–	1.68)	0.2276
≥ 3	1.67	(1.31	–	2.13)	0.0048
**Frequency of social contact with family**					
Less than once/w	1.00				
More than once/w	1.12	(0.99	–	1.27)	0.0650
**Frequency of social contact with friends**					
Less than once/w	1.00				
More than once/w	0.94	(0.83	–	1.08)	0.11 3806
**Frequency of social contact with neighbor**					
Less than once/w	1.00				
More than once/w	0.98	(0.87	–	1.11)	0.7673
**Reasons for suicidal ideation**					
Illness or disability	1.00				
Financial difficulties	0.65	(0.52	–	0.80)	<.0001
Loneliness	1.17	(0.95	–	1.44)	0.0253
Domestic troubles	1.36	(1.11	–	1.68)	<.0001
Troubles at work	1.01	(0.76	–	1.33)	0.0253
Others	0.99	(0.81	–	1.21)	<.0001
**Sex**					
Male	1.00				
Female	1.20	(1.03	–	1.41)	0.0206
**Age**					
19–29	1.00				
29–39	0.89	(0.71	–	1.11)	<.0001
40–49	0.84	(0.66	–	1.06)	<.0001
50–59	0.49	(0.38	–	0.63)	0.5858
60–69	0.50	(0.38	–	0.66)	0.7734
70 ~ 79	0.32	(0.23	–	0.44)	<.0001
≥ 80	0.16	(0.11	–	0.23)	<.0001
**Educational level**					
None/Middle school graduate	1.00				
High school graduate	1.18	(1.00	–	1.40)	0.0934
College graduate	0.92	(0.70	–	1.21)	0.0682
University graduate/higher	1.23	(0.98	–	1.54)	0.035
**Occupation**					
Others^b^	1.00				
Low-skilled blue collar	0.69	(0.57	–	0.83)	0.0035
High-skilled blue collar	1.04	(0.82	–	1.30)	0.0409
Low-skilled white collar	0.78	(0.63	–	0.95)	0.2352
High-skilled white collar	0.81	(0.65	–	1.00)	0.5467
**Marital status**					
W/o spouse	1.00				
With spouse	0.86	(0.73	–	1.01)	0.0599
**Number of household members**					
1	1.00				
2	1.21	(1.00	–	1.46)	0.0162
≥ 3	1.05	(0.85	–	1.30)	0.5762
**Region**					
Rural	1.00				
Urban	1.23	(1.06	–	1.43)	0.1228
Metropolitan	1.27	(1.08	–	1.50)	0.0328
**Household Income**					
Less than 1 million won/m	1.00				
1-2 mil won/m	0.98	(0.82	–	1.19)	0.6874
2-3 mil won/m	1.00	(0.81	–	1.24)	0.5117
3-4 mil won/m	0.84	(0.65	–	1.09)	0.0963
Over 4 mil won/m	0.98	(0.77	–	1.24)	0.7873
**Basic livelihood security recipient**					
Yes	1.00				
No	0.46	(0.38	–	0.56)	<.0001
**Depressive symptoms**					
Yes	1.00				
No	0.34	(0.30	–	0.38)	<.0001
**Perceived health status**					
Unhealthy	1.00				
Average	0.65	(0.56	–	0.75)	0.2861
Healthy	0.53	(0.44	–	0.65)	
**Difficulty in daily activity**					
Yes	1.00				0.0763
No	1.10	(0.93	–	1.30)	<.0001
**Currently Smoking**					
Yes	1.00				
No	0.94	(0.79	–	1.12)	0.4992
**High risk drinking**					
Yes	1.00				
No	1.20	(0.98	–	1.47)	0.0774

*OR* refers to odds ratio, *95% CI* refers to confidence interval

^a^ Number of social activities a respondent is currently participated in among religion, social, leisure and volunteer activities

^b^ Others include housewives, students, soldiers and unemployed

**Table 3:** Association between social participation and mental health consultation according to types of social activity

Variables	Mental health consultation				
	OR	95% CI			***P***
**Types of social activity**					
Leisure/sports (ref = no)	1.47	(1.25	–	1.73)	<.0001
Voluntary (ref = no)	1.13	(0.88	–	1.45)	0.3306
Religion (ref = no)	1.17	(1.02	–	1.33)	0.0119
Social (ref = no)	0.99	(0.86	–	1.15)	0.9385

*OR* refers to odds ratio, and *95% CI* refers to confidence interval

## Discussion

The study highlighted the link between social participation and the use of mental health consultation among individuals with suicidal ideation. There are increasing public concerns regarding mental health in Korea, yet many people are reluctant to use any form of mental health consultation services. Among various contributing factors, we attempted to focus on social participation and found that it was significantly related to the use of mental health consultation. Although a number of studies have demonstrated that social participation has beneficial health effects, inconsistent patterns have been found regarding its association with mental health consultation or service use. Some studies suggested that social participation acted as a substitute for the benefits of mental health services, thereby reducing the utilization of professional services [19]. Some studies found that social participation facilitated the utilization of professional services through advocacy or by decreasing any associated stigma and encouraging service use [17, 21–23].

In our study, participating in a higher number of social activities was positively and significantly associated with increased odds of utilizing mental health consultation services. Social participation has a role in both facilitating and nurturing interpersonal ties because they are carried out in the company of others [24] and thereby provide people with interactions with socially significant others [25]. Hence, these activities encompass all the beneficial components of social participation [24]. Of the four types of social activities, the association with religion led to utilization of mental health services among people who experienced suicidal ideation. Religious beliefs and participation in related activities are well-known determinants of mental health service utilization [26–28]. Whether or not religion has a positive or negative association, however, varies depending on an individual’s personal characteristics, such as the severity of any mental health problems, age, and community-level factors, such as the role of fellow community members.

Overall, social participation helps people to build supportive and trusting relationships, as well as a sense of communal integration, which are all necessary in developing beneficial social participation. In this study, we also included the frequency of contact, which is one measure to assess social networks. Our findings indicated that a person having more frequent contact with family may have more positive influences on mental health consultation than contact with neighbors or friends, although there was only a marginal significance. Earlier studies have found that various types of social networks may be qualitatively different and, therefore, have differential effects on a person in terms of their family, relatives, friends, and neighbors [18, 21, 22]. They suggested that family and relatives could have a more positive referral function on a person’s use of professional mental health services, as well as lowering the social stigmas associated with those services. In addition, living with a spouse increased the odds of utilizing services, which may also reflect the positive association of social support. Social networks with friends could be interpreted as providing relatively low social support when compared to family and, by extension, have fewer effects on respondents’ behaviors. Conversely, social contact with friends was found to possess a stress-reduction function and was therefore associated with reduced service utilization. Some studies suggested that, although social networks with friends may constitute comparatively weaker ties, they may still result in sharing of information about multiple services [29], which could then increase the use of alternative forms of healthcare interventions.

Regarding the reasons why respondents had suicidal ideation, those who thought of suicide due to financial difficulties were the least likely to use mental health consultation. Financial difficulties can have a severely negative impact of mental health and are a major risk factor that can lead to suicide. Since the main source of the problem is economical rather than clinical, they might have less need for professional consultations. Furthermore, they may experience greater unmet needs of mental health services due to a lack of affordability. Therefore, it is imperative that prevention strategies for those having financial problems should be differentiated from clinical approaches, for example, like providing welfare support or reducing unemployment [30].

In this study, we observed other covariates associated with mental health consultation. Depressive symptoms are a primary risk factor for suicidal behaviors and are also the strongest predictor of mental healthcare service utilization [31]. Our findings showed that men were less likely to use mental health services than women, which are consistent with the findings of previous studies on mental healthcare utilization [32, 33]. It is known that men and women have different help-seeking behaviors for a range of mental health issues [33, 34]. It then also becomes important to examine the potential differential effects of social networks on the help-seeking behaviors of women versus men. Alternatively, men and women may place different emphases on the importance of the beliefs and values of their respective social networks [34]. Additionally, our findings on consultation utilization due to suicidal ideation have been comparable to other studies and shown that older people who experience suicidal ideation are less likely to seek mental health treatment [33, 35, 36].

While the present study provides insight on the role of individuals’ social participation in relation to mental health consultation, the findings should be interpreted with caution owing to several limitations. First, this is a cross-sectional study, meaning that causality between two events cannot be distinguished. Social participation may buffer the experience of mental health problems, resulting in a decreased need for professional help [23]. In addition, it did not capture any changes in social participation and how those changes could be associated with the use of mental health consultation. Furthermore, the study did not assess whether an individual was participating in social activities at or around the time as their experience of suicidal ideation. An understanding of times frames for each variable would help gauge the extent to which retrospective recall biases may have operated. Future research with assessment for time frames and dynamics of social participation and how they are associated with suicidal ideation and mental health service use would provide a better understanding of behaviors of those who are at risk of suicide. Second, the use of mental health consultation and suicidal ideation were measured based on individuals’ self-report, and this could introduce recall bias of information in the study. Third, this study accounted for the quantitative aspect of social participation. Qualitative measures, including perceived social support, level of intimacy, and intensity were not assessed. In addition, this study did not distinguish between various types of mental health consultations. The effects of social participation may vary according to the types of services, such as general medical services, specialty psychiatric services, or other forms of services.

Despite these limitations, this study has several strengths. It involved a large, well-validated dataset collected from a nationally representative sample of the South Korean population. The study offers an understanding of social factors that are associated with mental health consultation.

## Conclusions

This study presented evidence of the links between social participation and mental health consultation in those with suicidal ideation. Based on the findings, the study suggests future investigation on the role of social capital such as participation, network, connectedness, and support and their various outcomes, especially in terms of the more subjective and qualitative concept of perceived support. Mental health and its related behaviors are complicated. Research from a social point of view will certainly widen the understanding of the dynamics of individuals’ mental health and related behaviors. Suicide prevention policies and programs designed to enhance social participation could potentially encourage people at risk of suicide to seek the help they need. Such evidence on social approaches can produce useful information to plan and implement comprehensive and effective strategies.

## Data Availability

The datasets generated and/or analyzed during the current study are available in the CHS website. (http://chs.cdc.go.kr).

## Abbreviations

OECD: Organization for Economic Co-operation and Development
KCHS: Korean Community Health Survey
KCDC: Korean Center for Disease Control and Prevention
